# Beverage-Induced Surface Changes in Biomimetic Dental Resin Composite: AFM and Bacterial Analysis

**DOI:** 10.1055/s-0044-1792009

**Published:** 2024-12-30

**Authors:** Rasha R. Basheer, Nermeen K. Hamza

**Affiliations:** 1 Department of Restorative Dentistry, Faculty of Dentistry, King Abdulaziz University, Jeddah, Saudi Arabia; 2 Department of Conservative Dentistry, Faculty of Dentistry, October University for Modern Sciences and Arts, Giza, Egypt

**Keywords:** supra-nanodental composite, energy drinks, sports drinks, surface roughness, bacterial adhesion, conservative dentistry

## Abstract

**Objective:**

Continuous advancements in composite resin materials have revolutionized and expanded its clinical use, improving its physical and mechanical properties. Attaining and retaining surface texture and gloss are crucial for the long-term durability of the composite resin material. This study investigated the supra-nanospherical filler composite material compared with different composite resin materials immersed in different beverages. The study evaluated their surface roughness and subsequent adhesion of bacteria.

**Materials and methods:**

A total of 144 specimens were made, using Teflon mold from different composite materials. Eighty-four specimens were used for surface roughness testing, using four different resin composite materials, Tetric N-Ceram (Ivoclar Vivadent, Schaan, Liechtenstein), Multichrome (Harvard Dental, Germany), Filtek Z350 XT (3M ESPE, Minnesota, United States), and Palfique LX5 (Tokuyama Dental Corporation, Taitō-Ku, Tokyo, Japan; *n* = 21). They were further subdivided into three subgroups according to the immersion solution ( *n* = 7) for Monster, Gatorade, and deionized water, which served as the control group. Surface roughness values were tested via atomic force microscopy (AFM). Then, for biofilm testing the bacterial count was performed on the remaining 60 composite specimens from the four tested composite materials ( *n* = 15), that were subdivided randomly based on the immersion solutions into three subgroups ( *n* = 5).

**Statistical analysis:**

Data were collected and statistically analyzed using the Kruskal–Wallis test followed by Dunn's post hoc test with Bonferroni's correction ( *p* ≤ 0.05). The intergroup comparison showed a significant difference among different composite materials ( *p* < 0.05), with the Multichrome showing the highest roughness values. Also, there was a significant difference between all composite materials with different beverages, with Palfique LX5 showing the lowest average roughness (Ra) values. All studied materials' average surface roughness, however, remained below the crucial Ra value of 0.2 μm. For the bacterial count, there was a significant difference between different materials in different beverages ( *p* < 0.05), with Z350 XT and Palfique LX5 showing the lowest bacterial count.

**Conclusion:**

Supra-nanospherical composite (Palfique LX5) exhibited better resistance to different beverage challenges regarding surface roughness, while nanohybrid composite (Z350) showed the least bacterial adherence.

## Introduction

Patients and dentists are becoming more interested in dental aesthetic restoration materials due to the increased emphasis on aesthetics in recent years. 1 As a result, high-quality restorative materials used in aesthetic restorative dentistry should withstand the harsh nature of the oral environment, including masticatory forces, pH fluctuations, and biofilm. 2 3 Hence, surface quality, including gloss and smoothness, is crucial for the aesthetic success of resin composite restorations. 4

Meenakshi et al stated that a perfect surface texture, dimensional integrity, sealing ability, and resistance to wear are requirements for ideal composite restorations. Also, they attributed the surface flaws larger than 0.2 μm to cause accumulation of plaque, cavities, and periodontal disease. 5 Prior studies have linked increased consumption of sugary drinks, such as orange juice, red wine, milk, coffee, and tea, to surface roughness. 6 7 Acidic beverages, such as Coca-Cola, pineapple juice, and citrus juice, can also change composite resins' physical characteristics and surface quality, impacting how well restorations function clinically. 8 9 10 In addition, newly introduced sports beverages provide nourishment and hydration, intending to improve athletic performance during physical activity. 11 12 However, they contain preservatives, acidulants, and high levels of carbohydrates (glucose, fructose, sucrose, and maltodextrin), which can cause an acidic oral environment after intake and alter the structure of composite resins. 11 13 Conversely, energy beverages have a low pH, and are made with sugar derivatives, B vitamins, taurine and carnitine amino acids, and herbal extracts including ginseng, guarana, and ginkgo biloba. 14 15

It is crucial to distinguish between sports and energy drinks 16 as caffeine is considered an active component in energy drinks. 17 Energy drinks contain caffeine, which does not enhance hydration. 18 It contains more carbohydrates than sports drinks. 9 Also, they are available in both sugary and sugar-free varieties. 11

Acidic diets can degrade resin-based restorative materials, leading to a weaker organic matrix and increased surface roughness. 19 Regular daily toothbrushing can remove superficial layers of the restoration and change the surface roughness. 13 19 This might lead to biofilm buildup on the teeth and diminish the brightness of restorations. 13 20 21 Hence, resin composite polymer matrix may hydrolyze, leading to deterioration in acidic pH solutions. 22 23

As a result, developing composite resin fillings is deemed difficult due to their heterogeneous nature, which results in rough surfaces after hardening. 24 Numerous factors, including monomer type, degree of curing, filler concentration, particle morphology and size, and bonding efficiency affect how rough a composite surface is. The best qualities of several distinct composites have recently been combined to create new types such as “nanofilled and supra-nanocomposites.” The nanoparticles that make up these novel restorative materials range in size from 1 to 100 nm. For these particles to endure the masticatory stresses encountered in the oral cavity, they must possess high physical and mechanical properties. These nanoparticles' superior polishing qualities and smooth surface quality are two of their advantages. The minuscule gaps that exist between inorganic particles known as nanoparticles are the cause of this. 25

Hence, a light-cured direct nanohybrid Filtek Z350 XT can be used for dental restorations on anterior and posterior teeth. 26 Its 5- 20-nm-sized zirconium and silica particles, or nanoclusters, offer superior durability against wear and gloss retention during polishing. 27

Conversely, a recently developed supra-nanospherical filler type of composite called Estelite Palfique LX5 has been shown to produce better surface texture and color stability. Resin composites incorporating supra-nanoparticles have the potential to reduce surface roughness due to their decreased susceptibility to detachment finishing by their polishing systems. 28 29 For instance, Palfique LX5 resin comprises 71% volume of silica–zirconium dioxide composite fillers, predominantly consisting of nanoparticles ranging in size from 0.1 to 0.3 μm. This gives the resin better wear resistance and less shrinkage during the polymerization, without sacrificing the ideal shine and polish. 29

The most common and traditional surface parameter is average roughness (Ra), known as center line average (CLA). 30 Ra represented the standard deviation (SD) of the integer divergence of roughness deviations from the median line over one sampled length, or the arithmetic average height. 31

There are two methods for measuring surface roughness: quantitative and qualitative. Atomic force microscopy (AFM) and surface profile assessment (e.g., stylus profilometer) are the two most often utilized quantitative techniques. 24 Since sports and energy drinks are widely gaining popularity, more data are needed to determine the impact of these drinks on dental restorations.

Thus, the objective of this *in vitro* study was to assess the supra-nanospherical filler composite material in comparison to different composite resin materials immersed in different beverages. Thus, their surface roughness and subsequent bacterial adhesion were evaluated. The null hypothesis was that there would be no difference between the supra-nanospherical filler composite, nanohybrid, and nanofilled composites regarding the surface roughness and bacterial adhesion.

## Materials and Methods

Four resin composite materials were tested, Tetric N-Ceram (Ivoclar Vivadent, Schaan, Liechtenstein), Multichrome (Harvard Dental, Berlin Germany), Filtek Z350 XT (3M ESPE, Minnesota, United States), and Palfique LX5 (Tokuyama Dental Corporation, Taitō-Ku, Tokyo, Japan; Table 1 ).

**Table 1:** List of the materials used

Dental composite material	Manufacturer's name	Fillers' classification	Filler composition	Filler content		Matrix composition	Shade
				wt%	vol%		
Tetric N-Ceram	Ivoclar Vivadent, Schaan, Liechtenstein	Nanohybrid composite	Nanohybrid, barium glass, prepolymer, ytterbium trifluoride, and mixed oxide	75–77	53–55	Dimethacrylate-based resins	A1
Multichrome	Harvard Dental, Berlin, Germany	Nanocomposite	Inorganic fillers < 0.2 µm	80	75	Dimethacrylate-based resins	A1
Filtek Z350 XT	3M ESPE, Minnesota, United States	Nanohybrid composite	Mix of; 20 nm silica fillers (nonagglomerated/nonaggregated), 4–11 nm zirconia fillers (nonagglomerated/nonaggregated), and aggregated zirconia/silica nanocluster comprising 20 nm silica and 4–11 nm zirconia particles	78.5	63.3	Bis-GMA/UDMA/TEGDMA, and Bis-EMA	A1
Palfique LX5	Tokuyama Dental Corporation, Taitō-Ku, Tokyo, Japan	Supra-nanocomposite	Silica–zirconia filler, with spherical morphology and an average inorganic particle size of 200 nm	82	71	Bis-GMA and TEGDMA monomers	A1

Abbreviations: Bis-EMA, bisphenol A ethyl dimethacrylate; Bis-GMA, bisphenol A glycol dimethacrylate; TEGDMA, triethylene glycol dimethacrylate; UDMA, urethane dimethacrylate.

### Sample Size Calculation

A power analysis was created with sufficient power to apply a statistical test of the null hypothesis—that there is no difference in surface roughness between the various tested groups. Using a power of 80%, an *α* level of 0.05, a *β* level of 0.2, and effect size ( *f* ) of 0.483 determined from the findings of a prior study, 32 the sample size ( *n* ) was determined to be a total of 84 samples, or 21 samples per group and 7 samples in each subgroup. To calculate the sample size, G*Power 3.1.9.7 was used.

Furthermore, a power analysis was planned to have sufficient power to apply a statistical test of the null hypothesis, which states that there should be no difference in biofilm retention between the several examined groups. With a power of 80%, an *α* level of 0.05, a *β* level of 0.2, and an effect size ( *f* ) of 0.583 determined by utilizing data from an earlier investigation, 33 the sample size ( *n* ) was determined to be 60 samples in total (15 samples per group and 5 samples each subgroup). G*Power 3.1.9.7 34 was used to calculate the sample size. 34

### Specimens Preparation

In total, 144 specimens were made, with 36 specimens from each material. This was achieved by utilizing a customized Teflon mold (2 mm in thickness and 5 mm in diameter), along with its copper ring. Each composite material was packed in the mold, with a Mylar strip and glass slides on top and bottom. Using a light-emitting diode (LED) curing device (LED device Mini LED, Satelec, Acteon, Viry-Châtillon, France) with a light intensity of 1,200 mW/cm ^2^ , and wavelength of 400 to 500 nm, all specimens were polymerized through the Mylar strip for 20 seconds (according to manufacturer instructions). The spectroradiometer (Demetron Research Corp., United States) was utilized to calibrate the intensity of the light. Subsequently, a caliper (Max Germany 6-inch SS) was used to check the specimens' dimensions. Then, Sof-Lex disks were used for polishing using a three-step procedure that involved applying medium, fine, and superfine grit in wet media for 15 seconds, each by the same operator for standardization. 26 After polishing, each specimen was washed and dried by air for 10 seconds. 26 All specimens were kept in distilled water at 37°C for 24 hours using an incubator. 35

### Immersion in Solutions and Grouping

Thirty-six specimens from each material were randomly distributed according to the test that will be performed as follows: for the surface roughness test, 21 specimens were divided according to immersion solutions into three subgroups ( *n* = 7) for Monster as an energy drink, Gatorade as a sports drink, and deionized water as control ( Table 2 ). Specimens underwent immersion in 25 mL for 5 seconds in the assigned solution, followed by immersion in 25 mL of artificial saliva for an additional 5 seconds, with this cycle repeated for a total of 24 cycles. 36 The specimens were then submerged in artificial saliva for 24 hours at a temperature of 37°C. For 14 days, this experimental cycle was repeated.

**Table 2:** Immersion solutions used

Trade name	Category	Components	Company name	pH
Monster Original	Energy drink	Carbonated water, sucrose, glucose, citric acid, natural flavors, taurine, sodium citrate, color added, panax ginseng root extract, L-carnitine, L-tartrate, caffeine, sorbic acid, benzoic acid, niacinamide, sodium chloride, *Glycine max* glucuronolactone, inositol, guarana seed extract, pyridoxine hydrochloride, sucralose, riboflavin, maltodextrin, and cyanocobalamin	Monster Beverages Cooperation, Corona, California, United States	2.7
Gatorade	Sports drink	Water, sucrose (table sugar), dextrose, citric acid, natural flavor, sodium chloride (table salt), sodium citrate, monopotassium phosphate, and flavoring/coloring ingredients. Some Gatorade flavor variations used to contain brominated vegetable oil as a stabilizer	The Gatorade Co., Chicago, Illinois, United States	2.9

Regarding the biofilm test, the remaining 15 specimens from each composite material, further subdivided according to the immersion solutions into 3 subgroups ( *n* = 5) for Monster, Gatorade, and distilled water as control.

### AFM Roughness Testing

All specimens were washed and blotted dry using absorbent paper 8 before the surface roughness test. An atomic force microscope (Model, VEECO Dimension 3100 Scanning Probe Microscope, Zurich, Germany) was utilized in contact mode, fitted with an auto probe head made by a Thermo-microscope. The Bruker Silicon Nitride Probe Model MLCT was utilized. With 512 × 512 data points and a scan rate of 1 Hz, the scan area was set to 10 × 10 µm ^2^ . IP 2.1 software was used for image analysis, and Pro-scan 1.8 software managed the scan parameters (Borregas Avenue, Sunnyvale, California, United States).

### *Streptococcus mutans* Preparation and Growth Method

*Streptococcus mutans* were cultured in sterilized Brain Heart Infusion (BHI) broth (HiMedia Laboratories Pvt. Ltd., Nashik, India), which included beef heart infusion, calf brain infusion, disodium hydrogen phosphate, glucose, peptone, and sodium chloride, with the final pH adjusted to 7.4. After incubating at 37°C for 24 hours, the culture reached a high growth concentration of approximately 106 CFU/mL. Colonies with similar morphology were isolated and grown in broth until the turbidity matched the 0.5 McFarland standard, yielding a suspension of approximately 1 to 2 × 108 CFU/mL. A susceptibility dilution test was then performed by creating serial dilutions, and aliquots of 25 μL from each dilution were then spread onto Mueller–Hinton agar plates. The number of colony-forming units (CFU) was determined after 24 hours of incubation. 37 38

### Statistical Analysis

Numerical data were represented as mean and SD values. The Shapiro–Wilk test was used to test for normality. The homogeneity of variances was tested using Levene's test. Data showed parametric distribution and variance homogeneity and were analyzed using two-way analysis of variance (ANOVA), followed by comparisons of simple main effects utilizing the error term of the ANOVA model with *p* -value adjustment using Dunn's post hoc test with Bonferroni's correction. The significance level was set at *p* < 0.05 within all tests. R statistical analysis software, version 4.3.2 for Windows, was used to conduct the analysis. 34

## Results

### Surface Roughness Results

**Fig. 1:**
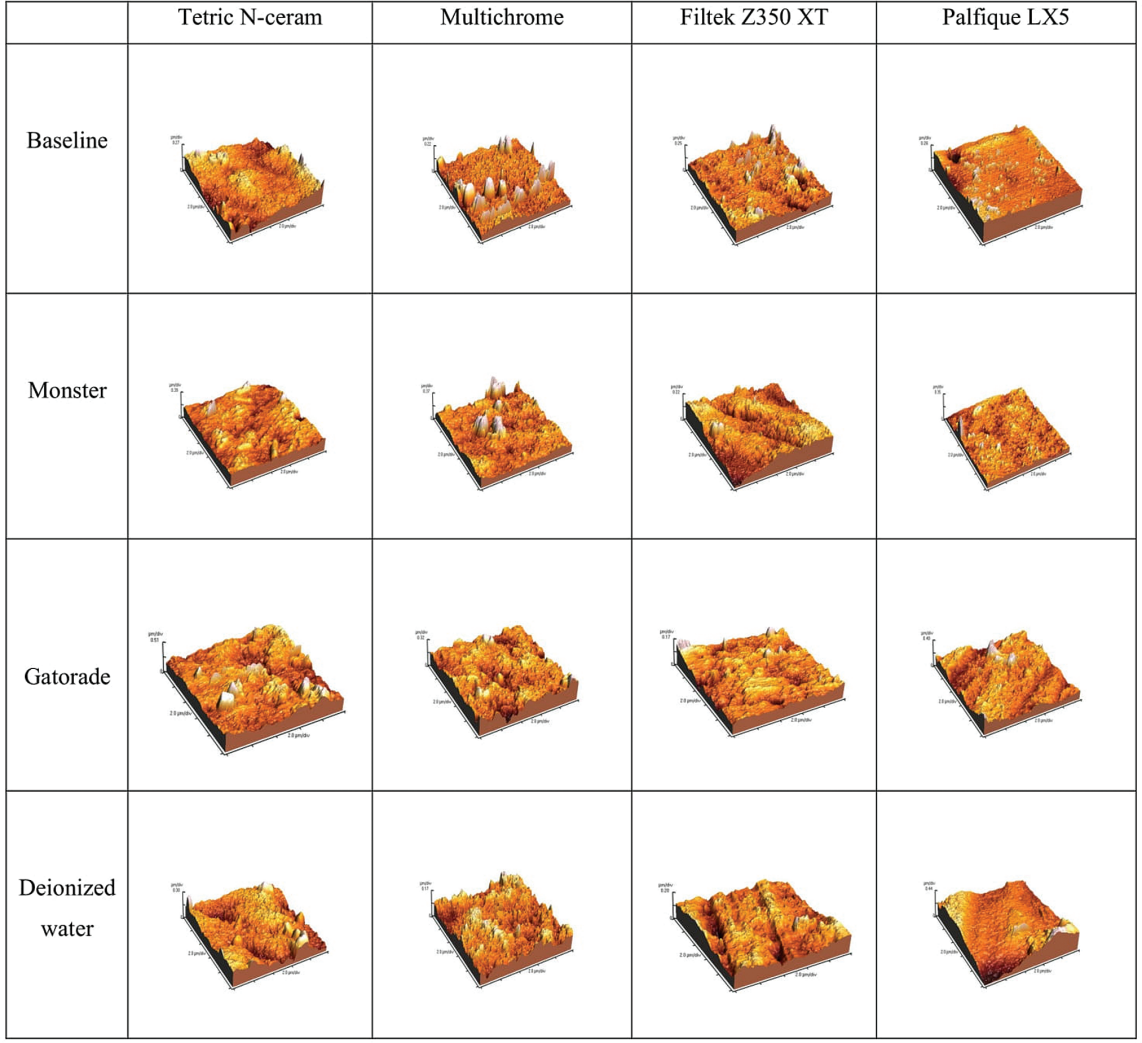
The 3D surface topography images for different composite material groups at baseline and after immersion in various beverages.

Table 3 shows a significant difference between all values measured in different restorative materials ( *p* < 0.05). Multichrome showed significantly higher values than Tetric N-Ceram and LX5 ( *p* < 0.05). In addition, they showed that Z350 XT had a significantly higher value than LX5 ( *p* < 0.05). According to the results, the values tested in various beverages varied significantly with baseline samples. Regarding the surface roughness measurements of different materials within different beverages, there was a significant difference between materials immersed in Monster and Gatorade ( *p* < 0.05; Fig. 1 ).

**Table 3:** Effect of materials and beverages on surface roughness

Measurement	Beverage	Mean ± standard deviation (SD)				*p* -value
		Tetric N-Ceram	Multichrome	Filtek Z350 XT	Palfique LX5	
Average roughness ( *Ra* )	Baseline	30.30 ± 5.11 ^Aba^	46.89 ± 6.25 ^Ab^	38.11 ± 8.10 ^ABb^	25.57 ± 6.83 ^Ba^	0.063
	Monster	49.52 ± 8.84 ^ABa^	40.86 ± 6.13 ^Bb^	63.91 ± 17.96 ^Aa^	45.73 ± 4.99 ^ABa^	0.029*
	Gatorade	42.36 ± 8.12 ^Ba^	72.90 ± 13.93 ^Aa^	48.64 ± 6.32 ^Bab^	29.77 ± 8.89 ^Ba^	< 0.001*
	Deionized water	41.95 ± 5.70 ^Aa^	61.04 ± 6.67 ^Aab^	52.86 ± 16.69 ^Aab^	40.24 ± 2.50 ^Aa^	0.052
	*p* -value	0.108	< 0.001*	0.017*	0.055	

Note: Values with different **upper- and lower-case** superscript letters within the same **horizontal row and vertical column** respectively are significantly different; *significant ( *p* < 0.05).

### Bacterial Count Results

**Fig. 2:**
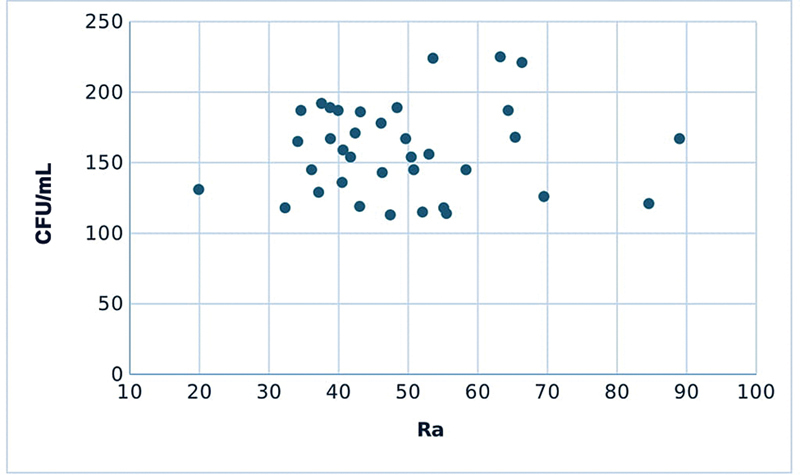
Scatter plot showing the correlation between Ra and bacterial count. Ra, average roughness.

Table 4 shows bacterial count results. There was a significant difference between values measured in different restorative materials ( *p* < 0.001), with the highest value measured in Multichrome, followed by Tetric N-Ceram, then LX5 and Z350 XT with the lowest value. All pairwise comparisons were statistically significant ( *p* < 0.001; Fig. 2 ). The effect of the interaction between surface roughness and bacterial count was not statistically significant ( *p* = 0.094).

**Table 4:** Effect of materials on bacterial count (CFU/mL)

Mean ± standard deviation (SD)				*p* -value
Tetric N-Ceram	Multichrome	Filtek Z350 XT	Palfique LX5	
169.44 ± 19.55 ^B^	189.56 ± 26.20 ^A^	125.22 ± 15.11 ^D^	150.33 ± 25.48 ^C^	<0.001*

Note: Values with different superscript letters within the same horizontal row are significantly different; *significant ( *p* < 0.05).

## Discussion

Restorations in the oral environment are subjected to several challenges, such as masticatory forces and different types of food and beverages. 39 40 One of the main challenges nowadays is the acidic beverages that have become popular in recent years, which cause erosive effects on composite restorations, leading to their failure by damaging their mechanical and aesthetic properties and even their surface integrity. 41 42 43 Therefore, to overcome these challenges, there is continuous development of resin composite. Modifications have been developed in resin matrix, filler particle size, and shape, leading to changes in its mechanical and physical properties. Supra-nanospherical filler composite was recently introduced, exhibiting higher clinical performance and color stability, with single shade allowing ease of manipulation and better finishing and polishing criteria. Surface roughness is considered the cornerstone of composite resin restoration success, and significantly affects aesthetics, surface properties, durability, and bacterial colonization. 40 Thus, the current study investigated the supra-nanospherical filler composite material compared to different composite resin materials (nanohybrid and nanofilled composites) immersed in different beverages, evaluating their surface roughness and subsequent adhesion of bacteria.

The AFM findings indicated notable variations among different materials following immersion in all beverages. Specifically, Multichrome exhibited significantly higher values than Tetric N-Ceram and Z350. LX5 demonstrated the lowest Ra values overall ( *p* < 0.05). Furthermore, each material displayed varying surface roughness depending on the beverage type: Multichrome showed the highest roughness after immersion in Gatorade, Z350 XT after immersion in Monster, and Palfique LX5 consistently displayed the lowest roughness across all beverage types tested. Regarding bacterial count results, Multichrome and Tetric N-Ceram showed higher bacterial count than Palfique LX5 and Z350 XT. Therefore, the null hypothesis was rejected, which stated that there would be no significant difference between the supra-nanospherical filler composite, nanohybrid, and nanofilled composites regarding the surface roughness and bacterial adhesion.

Despite the significant difference observed in Ra values between the baseline results and after immersion in different beverages, as well as the difference between various composite materials, the Ra values of all materials were below the critical roughness level (0.2 µm), and this was following El-Rashidy et al, who found out that after immersion of different composite materials in tea and red wine, the surface roughness did not exceed the critical value. 42 Meanwhile, they stated in their study that nanofilled composite showed superior physical and surface properties. Also, it was explained in other studies by the reduced spaces between the inorganic nanoclusters. 43 This was in contradiction with the results of the current study, as nanofilled composite (Multichrome) showed the highest Ra values among all composite materials used.

Furthermore, there was a contradiction between Camilotti et al and Kumari, as they found out that nanofilled composites showed a significantly higher surface roughness when immersed in carbonated drinks. 44 45 Also, it was stated that the surface roughness of Z350 XT (nanofilled composite) increased after 7 days but diminished over time when evaluated after 14 days, 46 and this was contradictory with the results of the present study. However, when they compared Z350 XT with Palfique LX5, the latter showed superior Ra values initially, after 7 days, and after 14 days, and this aligns with the results of the present study.

Several studies 47 48 49 50 have indicated that the performance of different composite materials can vary depending on their exposure to different types of food and beverages. This variability is primarily influenced by factors such as the type of resin utilized, the duration of exposure, and the interactions between the materials and the drinks. Moreover, differences in resin compositions and the presence of filler particles contribute to variations in surface irregularities and degradation when subjected to various foods and beverages.

Multichrome was the least impacted by immersion in Monster, while Z350 XT exhibited the highest Ra values. Conversely, Multichrome showed the greatest susceptibility to surface roughness when immersed in Gatorade, with Palfique LX5 being the least affected. Surface roughness variations could be due to different factors such as the pH of the energy/sports drinks, their staining agents, and titratable acidity, along with their interactions with different composite resin compositions, which influence their sorption and solubility properties. These factors may cause varying degrees of softening in the resin matrix or hydrolysis of the silane coupling agent, potentially leading to the dislodgment of filler particles and an increase in surface roughness. Additionally, the elution of unreacted monomers and the degrading effects on the polymer chains after exposure to different acidic solutions may also contribute to these changes. 50

Triethylene glycol dimethacrylate (TEGDMA) monomer is one of the main components of resin composite, which is added to reduce its viscosity; meanwhile, it increases its water sorption. Also, it is more prone to hydrolysis than bisphenol A glycol dimethacrylate (Bis-GMA) and bisphenol A ethyl dimethacrylate (Bis-EMA). 51 Z350 and LX5 contain TEGDMA; however, a significant difference in the Ra values was found among both materials after immersion in energy drinks, which might be referred to as the difference in the percentages of this monomer composition. 50 Cazzaniga et al and Bilgili et al stated that Palfique LX5 showed the greatest change in surface roughness when immersed in tea and distilled water, explaining that this might be due to the presence of TEGDMA, which has more affinity to water sorption, and this was contradicting with the results of the present study, as Palfique LX5 showed the lowest Ra values among all composite materials. 52 53

Additionally, water sorption of resin composite might be affected by the bond type, binding both the filler particles and the resin matrix, as well as the composition of the organic matrix. 54 The roughness observed in the AFM images following immersion in various beverages may be attributed to increased osmotic pressure at the interface between the filler and the matrix. 42 This pressure can lead to material hydrolysis, causing the expansion of monomers and their subsequent leaching, thereby contributing to surface roughness. 47 Additionally, water exhibits hydrolytic degradation properties that erode composite materials. This degradation involves hydroxyl ions attacking siloxane bonds, initiating hydrolytic degradation. This process is accompanied by matrix sorption, which creates pores and releases organic substances, ultimately altering surface properties. 41 55

Also, the increase in surface roughness after immersion in different beverages with different pH might be due to the chemicals from the acidic drinks leading to degradation of the restorative material, in which the pH of these drinks plays a crucial role in the corrosion of the materials. 11 Also, citric acid, as a main component of energy/sports drinks, is a strong inorganic acid that might cause organic matrix degradation. Furthermore, the ester groups of the dimethacrylate might undergo catalyzation favoring its hydrolysis, which increases the resin composite degradation. 48

According to the AFM images, as in Fig. 1 , LX5 showed an increase in the surface irregularities after immersion in different beverages, with Gatorade showing an increase in the peaks–valley distance than other beverages. Likewise, Tetric N-Ceram showed an increase in height differences. Furthermore, Multichrome showed the highest surface roughness of all materials after immersion in different beverages, with multiple surface irregularities and dark colors indicating several deep areas. Regarding images of the Z350 XT after immersion in different beverages, there is an increase in the difference between peak and valley, with dark color indicating severe irregularities.

There was a significant difference in bacterial counts among all tested materials. Multichrome and Tetric N-Ceram had the highest bacterial counts, while Palfique LX5 and Z350 XT had the lowest. A rough surface increases bacterial adherence, which might be due to its better protection against displacing forces, giving them the time to change into irreversible plaque formation. 51 Notable discrepancies were observed concerning the relationship between surface roughness and bacterial count, particularly with Z350 XT material. Despite Z350 XT exhibiting high average roughness values, it showed the lowest bacterial count among the tested materials. This unexpected result challenges the conventional assumption that higher surface roughness correlates with increased bacterial colonization. Furthermore, our findings also indicated a lack of significant correlation between bacterial count and surface roughness across the materials tested. These outcomes underscore the complexity of factors influencing bacterial adhesion and colonization on dental materials, suggesting that additional variables beyond surface roughness alone may play crucial roles in determining microbial adherence. These findings may be attributed to the low Ra values of all tested composite materials below the critical level. As stated earlier, 5 when Ra values are ≤0.2 μm, there is generally no significant correlation between surface roughness and bacterial adhesion. The findings of the current study were consistent with those of Demarco et al, 54 who stated that there is no obvious linkage between different Ra values and *S. mutans* adhesion. The results of the current study indicate that bacterial colonization might not depend solely on roughness measurements. These subtle distinctions highlight the complexity of interpreting surface roughness data about bacterial adhesion and confirm the importance of considering multiple factors influencing microbial behavior on dental materials.

Pellicle coating and initial bacterial adhesion, and biofilm formation are mainly influenced by the nature of the solid surface. A resin composite is considered a heterogeneous structure. The predominating monomer used in resin-based composite formulations has been Bis-GMA, which is frequently mixed with other dimethacrylates as TEGDMA, and urethane dimethacrylate (UDMA), as well as new monomers with increased molecular weight, which was developed to improve the mechanical and physical properties. 55 Several researches have stated that surface hydrophobicity plays an important role in determining materials' biological performance during the early stages of biofilm formation. 56 There are several debates regarding this point; several studies did not find a relationship between the hydrophobicity and microbiological adhesion to resin-based composite. 57 On the contrary, other studies claimed that the chemical composition of composite resin has an indirect link with the biofilm formation. Initial bacterial adhesion is promoted if bacteria and surfaces present similar hydrophobicity. 58 Furthermore, the duration of curing has an immediate connection with the biofilm formation, which is most likely related to the release of the unpolymerized monomer. 59

Kim et al 60 stated that Bis-GMA containing resin composite increased the hydrophobicity, favoring the adherence of *S. mutans* . However, in this study, Z350, LX5, and Tetric N-Ceram contain Bis-GMA, but there was a significant difference among all tested materials. Therefore, it might depend on the concentration of this high-viscosity monomer to other monomers. Also, Bis-GMA should not be considered the sole monomer contributing to the bacterial adherence properties. 61 Another explanation that might influence the bacterial adhesion is the filler particles size, shape, and amount. 62 Ikeda et al found that composite showed reduced bacterial adhesion when filler loading increases. 63 In the current study, the filler loading of the tested materials was approximately close, with LX5 showing the highest filler loading. Still, there was a significant difference in their bacterial count. Therefore, the speculation regarding the filler loading was in contradiction with the results of this study, while the particles' shape and size were different among various composite materials, which might be attributed to the significant difference in bacterial adhesion.

Several studies highlighted the significant role of surface properties in bacterial adhesion. The influence of surface free energy, particularly with surface roughness less than 0.2 μm, is crucial in understanding how materials interact with bacterial adhesion. As noted by Sengupta et al, 64 variations in filler composition can lead to different surface energy characteristics, impacting bacterial adherence. In the current study, Z350 and Palfique LX5 exhibited the lowest bacterial count, suggesting that their specific combination of silica and zirconia fillers might contribute to reduced bacterial adhesion. This contrasts with Tetric N-Ceram and Multichrome, which may possess different filler compositions or surface properties, that result in higher bacterial counts. Further investigation into the relationship between these materials' surface characteristics and their bacterial adhesion profiles would provide valuable insights into material selection for dental applications.

Although the present study aimed to replicate oral conditions, laboratory tests cannot perfectly simulate the intraoral environment. Thus, clinical trials are essential to validate the current findings. Future *in vitro* research should focus on better simulating the oral environment, particularly through the use of multispecies biofilm models. This approach will provide deeper insights into bacterial adhesion to resin-based materials and the interplay between microbial behavior and composite resin composition. Moreover, investigating additional factors influencing bacterial adhesion, such as surface free energy, hydrophobicity, and water sorption, is crucial. Identifying which components of composite resin play a key role in bacterial adhesion will also enhance our understanding. Finally, given the promising results from recent studies on antimicrobial techniques like ozone gas and ozonized water, 65 evaluating their effectiveness in reducing bacterial adhesion to resin-based materials could lead to innovative strategies for improving dental materials.

## Conclusion

Within the limitation of the present study, the supra-nanospherical filler composite (Palfique LX5) retained its surface finish and exhibited higher performance when subjected to different beverages' challenges, when compared with the nanofilled composite (Multichrome) and nanohybrid composite (Z350 XT and Tetric N-Ceram). However, all composite materials' surface roughness was below the critical Ra values. In addition, bacterial adherence was the lowest on the material surface of Palfique LX5 and Z350.

## Clinical Significance

Supra-nanocomposite and nanohybrid composite resin materials are recommended for aesthetic restorations. Also, sports and energy beverages might affect the composite material negatively.
